# Effects of Neutrophil Extracellular Traps in Patients With Septic Coagulopathy and Their Interaction With Autophagy

**DOI:** 10.3389/fimmu.2021.757041

**Published:** 2021-10-11

**Authors:** Jia-Yu Mao, Jia-Hui Zhang, Wei Cheng, Jian-Wei Chen, Na Cui

**Affiliations:** Department of Critical Care Medicine, State Key Laboratory of Complex Severe and Rare Diseases, Peking Union Medical College Hospital, Chinese Academy of Medical Science and Peking Union Medical College, Beijing, China

**Keywords:** sepsis, DIC, septic coagulopathy, NETs, autophagy

## Abstract

**Introduction:**

Neutrophil extracellular traps (NETs) act as a critical trigger of inflammation and coagulation. We hypothesized that NETs are associated with septic hypercoagulability.

**Materials and Methods:**

In total, 82 patients admitted with sepsis in the Department of Critical Care Medicine of Peking Union Medical College Hospital were enrolled between February 2017 and April 2018. Clinical and hematological parameters and thrombotic or hemorrhagic events were recorded. Blood samples were obtained to assess biomarkers of NET formation, including neutrophil elastase 2 (ELA2) and citrullinated histone H3, and endothelial-derived biomarker syndecan-1. Autophagy levels and their regulation pathway were also examined to explore their interaction with NETs.

**Result:**

Sepsis patients with disseminated intravascular coagulation (DIC) showed significantly higher levels of NET formation [ELA2, 1,247 (86–625) vs. 2,039 (1,544–2,534), p < 0.0001; H3, 140 (47–233) vs. 307 (199–415), p < 0.0001]. NET formation was independently associated with DIC risk [ELA2, OR 1.0028, 95% CI, 1.0010–1.0045; H3, OR 1.0104, 95% CI, 1.0032–1.0176] and mortality [ELA2, HR 1.0014, 95% CI, 1.0004–1.0024; H3, HR 1.0056, 95% CI, 1.0008–1.0115]. The area under the curve value for ELA2 in predicting DIC occurrence was 0.902 (95% CI, 0.816–0.957), and that of H3 was 0.870 (95% CI, 0.778–0.934). Furthermore, biomarkers of NET formation, endothelial cells, and autophagy exhibited a significant correlation [ELA2 and Syn (r = 0.5985, p < 0.0001), LC3B (r = −0.4224, p < 0.0001); H3 and Syn (r = 0.6383, p < 0.0001), LC3B (r = −0.3005, p = 0.0061)].

**Conclusion:**

Increased NET formation is significantly associated with sepsis-induced DIC incidence and mortality in sepsis patients, revealing a significant relationship with the autophagy pathway.

**Clinical Trial Registration:**

chictr.org.cn, identifier ChiCTR-ROC-17010750.

## Introduction

Sepsis is characterized by a broad inflammatory response and coagulation activation that both contribute to bacterial containment and lead to microangiopathy through uncontrolled thrombin and fibrin generation, potentially evolving toward disseminated intravascular coagulation (DIC) (1, 2). During sepsis, DIC exacerbates multiple organ dysfunction and increases the risk of death; thus, blood coagulation processes are a therapeutic target of interest (3). Nevertheless, pathophysiological mechanisms of DIC are still not fully understood.

Neutrophil extracellular traps (NETs) produced by activated neutrophils have recently been shown to provide a novel link between inflammation and thrombosis (4). NETs are portion of the innate immune response to microbial infections, consisting of cell-free DNA (cfDNA), histones, and neutrophil granule proteins (5). In accompany with red blood cells (RBCs), platelets, and fibrin, NET components promote formation of thrombi, especially in microvessels (6, 7). Moreover, cytokines released by neutrophils contribute to alteration of the membrane of vascular, especially endothelial cells (ECs), and therefore participate in sepsis-induced endothelial dysfunction and coagulation activation (8, 9).

Furthermore, the autophagy pathway plays an essential role in immunity through pathogen clearance mediated by immune cells, including macrophages and neutrophils (10). In particular, recent studies have revealed that autophagic activity is required for the formation and release of NETs (11). In this study, we explored the interaction between NETs and sepsis-induced DIC and its interaction with autophagy. We hypothesized that plasma NETs are associated with septic coagulopathy and mortality through the autophagy pathway.

## Materials and Methods

### Patients and Study Design

This prospective study was performed from April 2017 to April 2018 in the Department of Critical Care Medicine of Peking Union Medical College Hospital (PUMCH). The study was approved by the institutional review board of PUMCH (JS-1170). Informed consent was obtained from all enrolled patients. The study was registered at chictr.org.cn (identifier ChiCTR-OOB-17014129).

The inclusion criteria were as follows: 1) age ≥18 years, 2) intensive care unit (ICU) stay of more than 1 day, and 3) diagnosis of sepsis (see below). Patients who had basic hepatic or hematological disease were excluded. Sepsis was assessed according to the third international consensus definition as life-threatening organ dysfunction caused by a dysregulated host response to infection (12); organ dysfunction was defined as an acute change in total Sequential Organ Failure Assessment (SOFA) score of ≥2 points consequent to the infection. DIC was diagnosed if the International Society on Thrombosis and Haemostasis (ISTH) score was 5 or higher during the first 24 h of sepsis. The enrolled patients were divided into those without DIC, used as the control group, and those with DIC.

### Clinical and Laboratory Evaluation

Clinical history evaluation, laboratory tests, and plasma NET analysis were carried out within 24 h after ICU admission, including age, sex, coagulation parameters, blood chemistry, Acute Physiology and Chronic Health Evaluation (APACHE) II score, and SOFA score. Thrombotic events (defined as the number of patients diagnosed with deep-vein thrombosis pulmonary embolism, and coronary, cerebrovascular, or peripheral arterial thrombotic events), hemorrhagic events (defined as the number of patients diagnosed with hemoptysis, gastrointestinal hemorrhage, intracranial hemorrhage, or hematuria), and blood transfusion events (defined as the number of patients coming across RBC, plasma, or platelet transfusion) were collected. Follow-up data recorded included duration of ICU stay and 28-day mortality rate.

NETs consist of cfDNA, neutrophil granule proteins (like neutrophil elastase 2, myeloperoxidase (MPO), and cathepsin G) and nuclear protein (like histones H1, H2A, H2B, H3, and H4) (13). Levels of ELA2 (neutrophil elastase 2) and H3 (citrullinated histone H3) were measured to explore NET formation (14–16). Microtubule-associated protein light chain 3 type II (LC3II) was used as a marker of autophagy to visualize autophagosomes and to quantify autophagy in cells (17). As the upstream regulation pathway of autophagy, mammalian target of rapamycin (mTOR) and phosphorylated ribosome S6 protein kinase (PS6K) were measured (18). Blood samples were collected and centrifuged immediately; the plasma was withdrawn and stored at −80°C until assessment. Levels of ELA2, H3, syndecan-1, mTOR, PS6K, and LC3B concentrations were assessed by enzyme-linked immunosorbent assay (ELISA) (ELA2: abbexa abx196622, Beijing, China; H3: Cayman 501620, Shanghai, China; syndecan-1: CUSABIO CSB-E14983 h, Shanghai, China; mTOR: Abcam ab45996, Shanghai, China; PS6K: Abcam ab9366, Shanghai China; LC3B: Affinity AF4650, CST, China).

### Statistical Analysis

Normally distributed data were compared using Student’s t-test, and the results are expressed as the mean and standard deviation. Non-normally distributed data were analyzed with the Mann–Whitney U test and are presented as the median and interquartile intervals. Categorical variables were analyzed with the chi-square test and are recorded as proportions. Multivariate logistic regression was conducted to identify parameters for DIC risk prediction. Univariate logistic regression and the Cox proportional hazards survival model were applied to identify the independent contribution of prognostic factors to 28-day outcome prediction. The ability of NETs to predict the incidence of DIC risk was determined by receiver operating characteristic (ROC) curve analysis. Statistical analysis was performed using the SPSS 13.0 software (SPSS, Chicago, USA).

## Results

### Basic Characteristics

**Figure 1:**
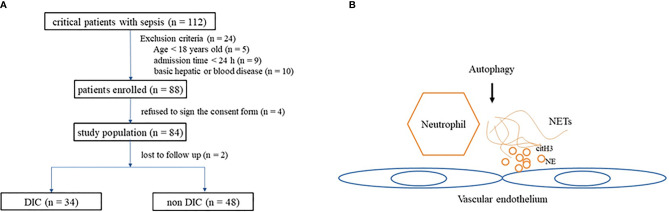
**(A)** Flowchart of patients included in the study. **(B)** Diagram depicting interaction of autophagy influence NETs and endothelium. NETs, neutrophil extracellular traps.

Within the study period (from April 2017 to April 2018), 112 patients diagnosed with sepsis were admitted. There were 24 patients who satisfied at least one of the exclusion criteria (nine were discharged 24 h after admission, 10 had basic hepatic or hematological disease, and five were <18 years old), four patients refused to sign the consent form, and two were lost to follow-up. A total of 82 patients were included in our study, and 34 among them met the DIC criteria (**Figure 1**).

**Figure 2:**
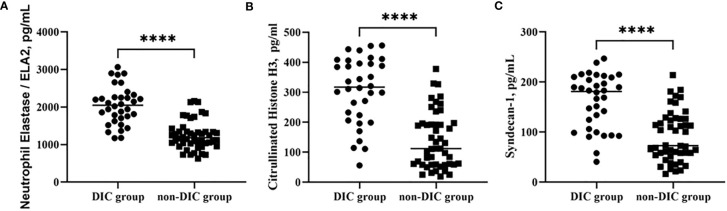
Comparison of NET levels, including ELA2 **(A)** and H3 **(B)**, and syndecan-1 **(C)** in groups of patients with or without DIC. ****p < 0.0001. NETs, neutrophil extracellular traps; DIC, disseminated intravascular coagulation.

**Table 1** shows the basic characteristics of all the patients after ICU admission. No significant difference was identified between the groups in age, though the majority of patients in the DIC group were male. Laboratory parameters of different groups are shown in **Table 2**. Overall, mortality, duration of ICU stay, APACHE II and SOFA scores, mechanical ventilation time, and renal replacement demand were significantly higher in the DIC group. The level of C-reactive protein, but not of procalcitonin, was significantly higher in the DIC group. In terms of coagulation indicators, DIC scores, prothrombin time, and D-dimer were higher in the DIC group, whereas plasma levels of platelets and fibrinogen were lower. Moreover, both thrombotic events and blood transfusion need were more frequent in the DIC group. Biomarkers of NET formation, including neutrophil elastase 2 (ELA2) [1,247 (869–1,625) vs. 2,039 (1,544–2,534), p < 0.0001^****^] and H3 [140 (47–233) vs. 307 (199–415), p < 0.0001^****^], showed significant differences between the groups. The endothelial-derived biomarker syndecan-1 was also higher [91 (42–141) vs. 162 (108–215), p < 0.0001^****^] in the DIC group, as shown in **Figure 2**.

**Table 1:** Baseline characteristics of the patients included.

Characteristics	Non-DIC, n = 48	DIC, n = 34	p
Age, years	66.9 (52.1–81.6)	63.5 (54.0–76.0)	0.0734
Sex, n (%)			
Male	26 (54.2)	23 (67.6)	0.2583
Basic disease			
Coronary heart disease	6 (12.5)	6 (17.6)	0.5415
Arrhythmia	5 (10.4)	3 (8.8)	0.9999
Valvular lesions	4 (8.3)	0 (0)	0.1378
Gastrointestinal cancer	6 (12.5)	3 (8.8)	0.7290
Gallstone	5 (10.4)	1 (2.9)	0.3927
Chronic kidney disease	3 (6.25)	1 (2.9)	0.6382
Cerebrovascular disease	8 (16.7)	3 (8.8)	0.3478
Infection site, n (%)			
Bloodstream	12 (25)	6 (17.6)	0.4281
Lung	32 (66.7)	24 (70.6)	0.7069
Abdominal cavity	6 (12.5)	11 (32.4)	0.0508
Bile duct	3 (6.3)	1 (2.9)	0.6382
Soft tissue	1 (2.1)	4 (11.8)	0.1545
Urinary tract infection	2 (4.2)	1 (2.9)	0.9999
Pathogen			
Gram-negative bacilli	38 (79.2)	25 (73.5)	0.6017
Gram-positive coccus	16 (33.3)	15 (44.1)	0.3612
Gram-positive bacillus	5 (10.4)	1 (2.9)	0.3927
Fungus	5 (10.4)	6 (17.6)	0.5123
Virus	4 (8.3)	3 (8.8)	0.9999

DIC, disseminated intravascular coagulation.

**Table 2:** Laboratory parameters in different groups.

Characteristics	Non-DIC, n = 48	DIC, n = 34	p
28-day mortality, n (%)	3 (6.3)	17 (50)	<0.0001^****^
ICU stay time, days	11.3 (3.2–19.4)	15.3 (7.2–23.3)	0.0350^*^
APACHE II	16.7 (11.1–22.3)	20.4 (13.4–27.5)	0.0107^*^
SOFA	8.0 (4.1–11.9)	11.2 (7.2–15.2)	0.0005^***^
MV duration	81 (0–92)	157 (75–218)	0.0009^***^
CRRT	3 (6.3)	8 (23.5)	0.0237^*^
PCT	2.0 (0.5–7.3)	5.0 (2.9–21.0)	0.34
CRP	145.2 (36.8–187.3)	264.6 (125.1–351.1)	<0.0001^****^
DIC score	3.0 (2.0–4.0)	5.0 (5.0–7.0)	<0.0001^****^
PLT, *10^9^/L	170.9 (59.7–282.1)	73.7 (24.3–123.1)	<0.0001^****^
PT, s	17.0 (10.8–23.3)	22.9 (11.9–34.0)	0.0031^**^
Fbg, g/L	3.8 (2.2–5.4)	2.8 (1.6–3.9)	0.0015^**^
D-Dimer, mg/L FEU	4.3 (2.3–8.7)	16.9 (8.3–23.2)	0.0002^***^
Thrombotic events	9 (18.8)	15 (44)	0.0156^*^
Hemorrhagic events	7 (14.6)	11 (32.4)	0.0643
Blood transfusion			
RBC	23 (47.9)	24 (70.6)	0.0456^*^
Plasma	16 (33.3)	27 (79.4)	<0.0001^****^
PLT	4 (8.3)	10 (29.4)	0.0174^*^
ELA2, pg/ml	1,247 (869–1,625)	2,039 (1,544–2,534)	<0.0001^****^
H3, pg/ml	140 (47–233)	307 (199–415)	<0.0001^****^
Syn, pg/ml	91 (42–141)	162 (108–215)	<0.0001^****^

DIC, disseminated intravascular coagulation; ICU, intensive care unit; APACHE, Acute Physiology and Chronic Health Evaluation; SOFA, Sequential Organ Failure Assessment; MV, mechanical ventilation; CRRT, continuous renal replacement therapy; PCT, procalcitonin; CRP, C-reactive protein; PLT, platelet; PT, prothrombin time; Fbg, fibrinogen; RBC, red blood cell; ELA2, neutrophil elastase 2; H3, citrullinated histone H3; Syn, syndecan-1.

*p < 0.05, **p < 0.01, ***p < 0.001, ****p < 0.0001.

### Risk Factors for Disseminated Intravascular Coagulation Risk in Sepsis Patients

**Figure 3:**
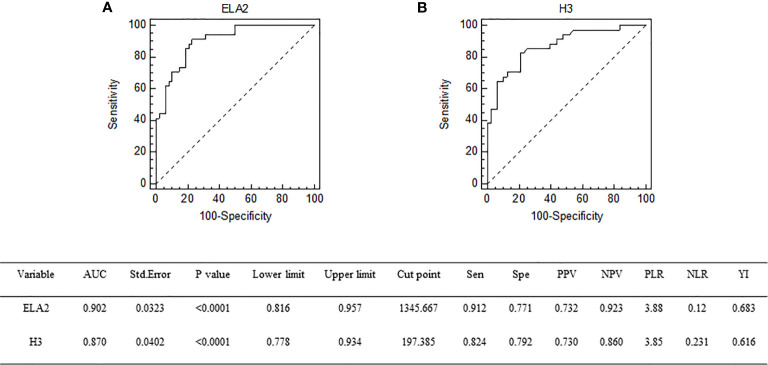
Receiver operating characteristic curve of ELA2 **(A)** and H3 **(B)** for prediction of DIC occurrence. DIC, disseminated intravascular coagulation.

Univariate logistic regression analysis was conducted to identify variables associated with DIC in sepsis patients; and among the variables, APACHE II score, SOFA score, ELA2, and H3 were found to be associated with DIC. Therefore, these variables were taken into multivariate logistic regression for adjustment, the OR for DIC of ELA2 was 1.0028 (95% CI, 1.0010–1.0045, p = 0.0023), and H3 was 1.0104 (95% CI, 1.0032–1.0176, p = 0.0046) after adjustment for risk factors (**Tables 3** and **4**). A ROC curve for DIC occurrence was drawn (**Figure 3**), and the area under the curve for ELA2 and H3 was 0.902 (95% CI, 0.816–0.957, p < 0.0001) and 0.870 (95% CI, 0.778–0.934, p < 0.0001), respectively.

**Table 3:** Univariate logistic regression analysis for possible risk factors for DIC.

Variable	B	SE	p	OR	95% CI for OR	
					Lower	Upper
Age	−0.0300	0.0169	0.0770	0.9705	0.9388	1.0033
APACHE II	0.0942	0.0391	0.0160	1.0988	1.0177	1.1863
SOFA	0.2031	0.0636	0.0014	1.2252	1.0817	1.3878
ELA2	0.0038	0.0008	<0.0001	1.0038	1.0022	1.0053
H3	0.0138	0.0029	<0.0001	1.0139	1.0082	1.0197

DIC, disseminated intravascular coagulation; APACHE, Acute Physiology and Chronic Health Evaluation; SOFA, Sequential Organ Failure Assessment; ELA2, neutrophil elastase 2; H3, citrullinated histone H3.

**Table 4:** Multivariate logistic regression analysis for possible risk factors for DIC.

Variable	B	SE	p	OR	95% CI for OR	
					Lower	Upper
APACHE II	−0.0045	0.0659	0.9455	0.9955	0.8748	1.1328
SOFA	0.2278	0.1063	0.0320	1.2559	1.0197	1.5467
ELA2	0.0028	0.0009	0.0023	1.0028	1.0010	1.0045
H3	0.0103	0.0036	0.0046	1.0104	1.0032	1.0176

DIC, disseminated intravascular coagulation; APACHE, Acute Physiology and Chronic Health Evaluation; SOFA, Sequential Organ Failure Assessment; ELA2, neutrophil elastase 2; H3, citrullinated histone H3.

The association between NETs and clinical outcomes like thrombotic and bleeding events was also conducted through univariate logistic regression analysis. The OR of ELA2 for thrombotic events was 1.0010 (95% CI, 1.0002–1.0019, p = 0.0149), and that for bleeding events was 1.0012 (95% CI, 1.0003–1.0022, p = 0.0072); the OR of H3 for thrombotic events was 1.0040 (95% CI, 1.0001–1.0078, p = 0.0374), and that for bleeding events was 1.0046 (95% CI, 1.0003–1.0088, p = 0.0293).

### Risk Factors for 28-Day Mortality in Sepsis Patients

**Figure 4:**
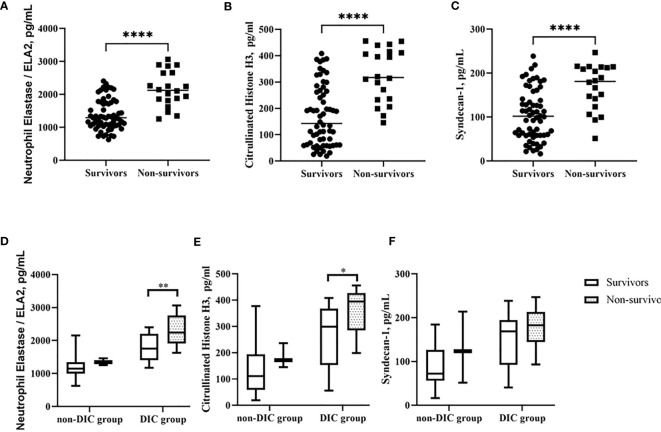
Comparison of NETs level in different groups. **(A–C)** ELA2, H3 and Syndecan-1 levels in groups of survivor and non-survivor patients. **(D–F)** ELA2, H3 and Syndecan-1 levels in groups of patients with or without DIC stratified by 28-day mortality. *p < 0.05, **p < 0.01, ****p < 0.0001.

Next, we compared the features conducted by 28-day mortality and found ELA2 [1,386 (928–1,845) vs. 2,162 (1,636–2,687), p < 0.0001], H3 [172 (57–286) vs. 326 (226–426), p < 0.0001], and Syn [105 (48–162) vs. 168 (117–219), p < 0.0001] to be significantly lower in the survivor group (**Figures 4A**–**C**). Levels of these biomarkers were evaluated after stratification according to 28-day mortality in the DIC and non-DIC groups, and the same trend was observed, especially in the former (**Figures 4D**–**F**).

Univariate logistic regression analysis indicated that the variables SOFA score, ELA2, and H3 level were associated with mortality in patients with sepsis. Additionally, a Cox proportional hazards survival model was conducted to adjust relevant variables for a poor prognosis; the HR of ELA2 was 1.0014 (95% CI, 1.0004–1.0024; p = 0.0055), and that of H3 was 1.0056 (95% CI, 1.0008–1.0105, p = 0.0223) (**Tables 5** and **6**).

**Table 5:** Univariate logistic regression analysis for possible risk factors for prognosis.

Variable	B	SE	p	OR	95% CI for OR	
					Lower	Upper
Age	−0.0035	0.0185	0.8493	0.9965	0.9609	1.0334
SOFA	0.2229	0.0757	0.0032	1.2497	1.0774	1.4496
ELA2	0.0029	0.0007	<0.0001	1.0029	1.0015	1.0042
H3	0.0113	0.0028	<0.0001	1.0113	1.0058	1.0169

SOFA, Sequential Organ Failure Assessment; ELA2, neutrophil elastase 2; H3, citrullinated histone H3.

**Table 6:** Cox regression analysis for possible risk factors for prognosis.

Variable	B	SE	p	HR	95% CI for OR	
					Lower	Upper
Age	0.0088	0.0212	0.6765	1.0089	0.9681	1.0514
SOFA	0.1475	0.0735	0.0449	1.1590	1.0041	1.3377
ELA2	0.0014	0.0005	0.0055	1.0014	1.0004	1.0025
H3	0.0056	0.0025	0.0223	1.0056	1.0008	1.0105

SOFA, Sequential Organ Failure Assessment; ELA2, neutrophil elastase 2; H3, citrullinated histone H3.

### Biomarkers of Autophagy in the Disseminated Intravascular Coagulation Group

**Figure 5:**
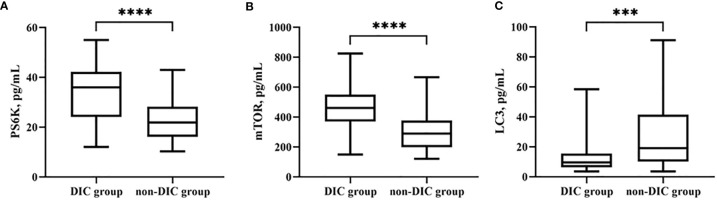
Comparison of autophagy biomarkers levels, including PS6K **(A)**, mTOR **(B)**, and LC3B **(C)** in groups of patients with or without DIC. ***p < 0.001, ****p < 0.0001. DIC, disseminated intravascular coagulation.

Biomarkers of autophagy were also examined. LC3B [30.7 (5.0–56.5) vs. 13.3 (2.1–24.5), p = 0.0004***] was significantly lower in the DIC group. Among upstream pathways, mTOR [295.3 (180.4–410.1) vs. 477.2 (323.1–631.3), p < 0.0001****] and PS6K [22.7 (14.8–30.5) vs. 33.7 (21.9–45.4), p < 0.0001****] levels were higher in patients with DIC (shown in **Table 7** and **Figure 5**).

**Table 7:** Levels of biomarkers of autophagy in different groups.

Characteristics	Non-DIC, n = 48	DIC, n = 34	p
LC3B, pg/ml	30.7 (5.0–56.5)	13.3 (2.1–24.5)	0.0004^***^
mTOR, pg/ml PS6K, pg/ml	295.3 (180.4–410.1) 22.7 (14.8–30.5)	477.2 (323.1–631.3) 33.7 (21.9–45.4)	<0.0001^****^ <0.0001^****^

DIC, disseminated intravascular coagulation; LC3B, microtubule-associated protein light chain 3 type II; mTOR, mammalian target of rapamycin; PS6K, phosphorylated ribosome S6 protein kinase.

***p < 0.001, ****p < 0.0001.

### Relationships Among Neutrophil Extracellular Traps, Endothelial Markers, and Autophagy

**Figure 6:**
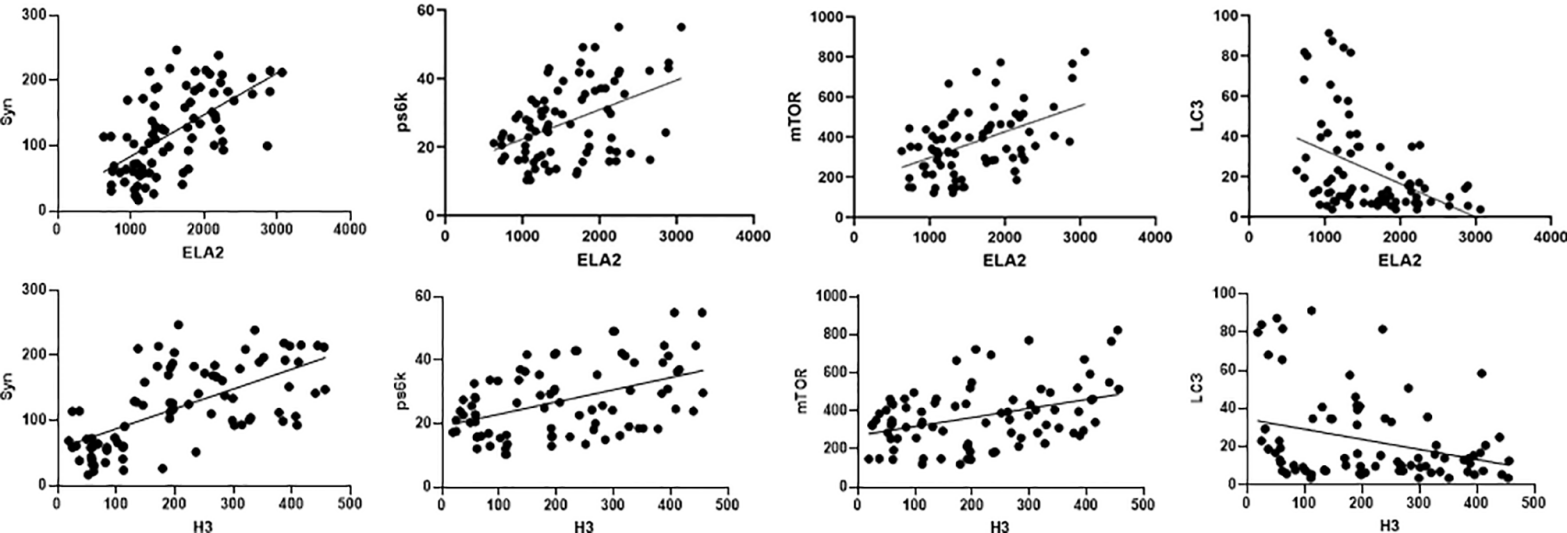
Correlations between NET level (including ELA2 and H3), syndecan-1, and autophagy biomarkers (including PS6K, mTOR, and LC3B) in patients with sepsis.

We hypothesized that NETs, endothelial markers, and autophagy interact closely in the mechanism of DIC. Pearson’s analysis was used to identify significant correlations between levels of ELA2 and Syn (r = 0.5985, p < 0.0001), PS6K (r = 0.4505, p < 0.0001), mTOR (r = 0.4708, p < 0.0001), LC3B (r = −0.4224, p < 0.0001), H3 and Syn (r = 0.6383, p < 0.0001), PS6K (r = 0.4506, p < 0.0001), mTOR (r = 0.3841, p = 0.0004), and LC3B (r = −0.3005, p = 0.0061) (**Figure 6**).

## Discussion

The present study reveals the first view of circulating NET formation in sepsis patients diagnosed with DIC. To explore the effect of NETs in septic coagulopathy, we investigated levels of plasma NET formation in sepsis patients with DIC, revealing higher plasma NET formation [ELA2, 1,247 (869–1,625) vs. 2,039 (1,544–2,534), p < 0.0001; H3, 140 (47–233) vs. 307 (199–415), p < 0.0001] than in control group patients. Indeed, NET formation was independently associated with DIC risk (ELA2, OR 1.0028, 95% CI, 1.0010–1.0045; H3, OR 1.0104, 95% CI, 1.0032–1.0176). The value drawn by ROC curve for ELA2 in predicting DIC occurrence was 0.902 (95% CI, 0.816–0.957); that of H3 was 0.870 (95% CI, 0.778–0.934, p < 0.0001). Sepsis patients with DIC had a higher prothrombin time, D-dimer levels, and DIC scores and lower levels of platelets and fibrinogen. Thrombotic events and blood transfusion need were also more frequent in the DIC group. NET formation was also independently associated with both thrombotic and bleeding events risk. Overall, sepsis patients with DIC had worse outcomes and experienced longer ICU stay times, more severe organ dysfunction, worse respiratory conditions, and frequent renal replacement therapy. NET formation [ELA2, HR 1.0014, 95% CI, 1.0004–1.0024; H3, HR 1.0056, 95% CI, 1.0008–1.0115] was independently associated with mortality.

To our knowledge, our research constitutes the first prospective clinical study of NET formation in sepsis patients with DIC. In recent years, researchers have proposed the existence of an effective mechanism involved in the crosstalk between blood coagulation and the immune response that result in formation of immunothrombosis (19). Patients with sepsis commonly experience coagulation dysfunction and have a poor prognosis. In the body’s innate immunity, neutrophils play a key role in response to infection. NETs eliminate pathogens through neutrophils, but the imbalance also causes tissue damage. Thus, exploring the effect of NETs is essential for the diagnosis and prognosis of septic coagulopathy. However, only few studies to date explored the role of NETs in septic coagulopathy in clinical research. Yang et al. showed that during sepsis, systemic inflammation promotes neutrophils to release NETs, increasing risk of venous thromboembolism (16), and Stiel et al. first visualized circulating NETs using cell fluorescence during septic shock-induced DIC in a small sample study (20). In our study, we first revealed that sepsis patients with DIC have higher plasma NET formation, that NET formation are independently associated with DIC risk and mortality, and that serum concentrations of NET formation are able to predict incidence of DIC during sepsis. These findings confirm the role of NETs in septic coagulopathy, piquing our interest in the underlying mechanism. In a variety of sepsis mouse models, NETs induced intravascular coagulation results in microvascular dysfunction (21, 22). These findings are meaningful and deserve our further exploration.

The endothelium plays an essential role in hemostasis balance; endothelial damage is a critical trigger on coagulopathy in severe sepsis (23). Glycocalyx damage, leading to a higher amount of circulating syndecan-1 (24), can range from disturbances of luminal layer to excessive destruction and degradation of the entire glycocalyx. Therefore, circulating syndecan-1 was measured in sepsis patients in our study. Consistent with previous studies (25, 26), we found that syndecan-1 was significantly increased in the group of sepsis patients with DIC. As expected, NET formation showed a close relationship with syndecan-1 (ELA2 vs. Syn, r = 0.5985, p < 0.0001; H3 vs. Syn, r = 0.6383, p < 0.0001). Neutrophil–ECs engage in close crosstalk, and ECs lead to increased NET formation; moreover, prolonged coculture of neutrophils with ECs results in EC damage (13). In septic-infected mice, NETs induce ECs to release adhesion factors and tissue factor (TF) and then to recruit inflammatory cells and facilitate thrombosis (27). Thus, neutrophil–EC interactions play an essential role in the formation and development of thrombi in sepsis.

A new discovery of the relationship between NETs and autophagy has attracted much interest. Autophagy is a vital cellular mechanism in regulation of cell homeostasis and survival (28). Interaction between autophagy and NETs was firstly explored by Remijsen et al. (10), who proposed NET formation requires both autophagy and superoxide generation. Therefore, biomarkers of autophagy were examined in our study, and we found that the serum level of LC3B, a major autophagy protein, was lower in the DIC group. Furthermore, levels of components of the mTOR and PS6K pathways, upstream of autophagy, were significantly higher in patients with DIC. We also discovered that NET formation exhibits a significant relationship with autophagy markers, as shown by ELA2 and LC3B (r = −0.4224, p < 0.0001) and H3 and LC3B (r = −0.3005, p = 0.0061). In the context of autoimmune diseases like systemic lupus erythematosus (SLE) or rheumatoid arthritis, pathological roles of NETs have already been acknowledged (29, 30). Autophagy not only participates in NET formation but also inhibits excess NET release (31). We hypothesize that the autophagy pathway may participate in NET formation. Future studies are needed to reveal the missing links explaining the complex set of regulatory mechanisms.

Our study had several limitations. This was a single-center study, which may limit generalizability. A larger cohort or multicenter studies and subgroup analysis should be conducted. Second, NETs can be detected by serum assays or flow cytometry; flow cytometry assays are more specific than serum markers but are more difficult and may result in measurement variability and interpretation biases (32). Third, blood samples were only collected within 24 h of patient admission; a dynamic analysis of NETs may be worthy of further study. Finally, more samples of control group like normal people or postsurgical condition may help to further clarify effect of NETs during sepsis.

## Conclusions

Increased NET formation was significantly associated with DIC incidence and 28-day mortality in sepsis patients, demonstrating a significant relationship with the autophagy pathway.

## Data Availability Statement

The raw data supporting the conclusions of this article will be made available by the authors, without undue reservation.

## Ethics Statement

The studies involving human participants were reviewed and approved by ethics committee of Peking Union Medical College Hospital, Beijing, China (Approval No. JS-1170). The patients/participants provided their written informed consent to participate in this study.

## Author Contributions

CN designed the experiment. MJY drafted and revise the manuscript. CJW analyzed and assembled input data. CW participated in the design of the study and the acquisition of data. ZJH conceived of the study and helped to draft the manuscript. All authors contributed to the article and approved the submitted version.

## Funding

The work was supported by the National Natural Science Foundation of China (No. 82072226, No. 81601657), Beijing Municipal Science and Technology Commission (No. Z201100005520049), Non-profit Central Research Institute Fund of Chinese Academy of Medical Sciences (No. 2019XK320040), Tibet Natural Science Foundation (No. XZ2019ZR-ZY12(Z)), and Excellence Program of Key Clinical Specialty of Beijing in 2020 (No. ZK128001).

## Conflict of Interest

The authors declare that the research was conducted in the absence of any commercial or financial relationships that could be construed as a potential conflict of interest.

## Publisher’s Note

All claims expressed in this article are solely those of the authors and do not necessarily represent those of their affiliated organizations, or those of the publisher, the editors and the reviewers. Any product that may be evaluated in this article, or claim that may be made by its manufacturer, is not guaranteed or endorsed by the publisher.

## Abbreviations

NETs, neutrophil extracellular traps; ELA2, neutrophil elastase 2; H3, citrullinated histone H3; DIC, disseminated intravascular coagulation; cfDNA, cell-free DNA; PUMCH, Peking Union Medical College Hospital; ICU, intensive care unit; SOFA, Sequential Organ Failure Assessment; ISTH, International Society on Thrombosis and Haemostasis; APACHE, Acute Physiology and Chronic Health Evaluation; mTOR, mammalian target of rapamycin; PS6K, phosphorylated ribosome S6 protein kinase; LC3B, microtubule-associated protein light chain 3 type II; ELISA, enzyme-linked immunosorbent assay; OR, odds ratio; HR, hazard ratio; ROC, receiver operating characteristic; EC, endothelial cell; TF, tissue factor; SLE, systemic lupus erythematosus; MV, mechanical ventilation; CRRT, continuous renal replacement therapy; PCT, procalcitonin; CRP, C-reactive protein; PLT, platelet; PT, prothrombin time; Fbg, fibrinogen; RBC, red blood cell; Syn, syndecan-1.
